# An up-dated meta-analysis of major adverse cardiac events on triple versus dual antiplatelet therapy after percutaneous coronary intervention in patients with type 2 diabetes mellitus

**DOI:** 10.1016/j.dib.2018.06.091

**Published:** 2018-06-30

**Authors:** Mao Liu, Wenjing Lu, Ling Chen, Yanmei Wang, Zhuoling Zhao, Mei Zhang, Weiwei Du, Zhan Lv

**Affiliations:** a Department of Cardiology, the Affiliated Hospital of North Sichuan Medical College, Nanchong 637000, PR China; b Cardiovascular Research Center, the Affiliated Hospital of North Sichuan Medical College, Nanchong, Sichuan Province, PR China; c Department of Cardiology, the Fifth Affiliated Hospital of Sun Yat-sen University, Zhuhai 519000, PR China; d Department of Clinical Medicine, Yaan Polytechnic College, Yaan 625000, PR China

## Abstract

This meta-analysis is conducted to assess the efficiency and safety of triple antiplatelet therapy in patients with type 2 diabetes mellitus (T2DM) who have received coronary stents implantation. The risk of major adverse cardiac events (MACEs), target vessel revascularization (TVR), target lesion revascularization (TLR), myocardial infarction (MI) and bleeding events were evaluated in this meta-analysis. Eight randomized controlled trials incorporating 1700 participants were included. During a follow-up of 12 months after stents implantation, the risk of TVR, TLR and MACEs in Triple group were lower than that of Dual group. There was no significant difference in the comparison of stent thrombosis and bleeding events between the two groups. Triple antiplatelet therapy is effective in reducing adverse cardiovascular outcomes in T2DM patients after stents implantation, without increasing the risk of bleeding events. Advanced designed and large-scale trails are deserved in the future.

**Specifications Table**

**Table:** 

Subject area	*Medicine*
More specific subject area	*Cardiology*
Type of data	*Table and figure*
How data was acquired	*Systematic review with Review Manager 5.2*
Data format	*Analyzed*
Experimental factors	*None*
Experimental features	*None*
Data source location	*NA*
Data accessibility	*Data are within this article*

**Value of the data**

• High quality data cover eight randomized clinical trials.
• Provide EBM data concerning triple antiplatelet therapy in type 2 diabetes patients.
• Data is helpful for the clinical therapy of patients with type 2 diabetes and coronary artery disease.
• Researchers or physicians can use our data for analysis or clinical report.

## 1 Data

Dual antiplatelet therapy consisting of aspirin and clopidogrel is a cornerstone of management for coronary artery disease (CAD) patients, especially for those who have received stents implantation. Previous studies have found that addition of cilostazol was an effective and relatively safe strategy in preventing major adverse cardiac events (MACEs) in type 2 diabetes mellitus (T2DM) patients. The value of triple antiplatelet therapy had not been well proved. Therefore, this meta-analysis was conducted to systematically evaluate the efficiency and safety of this strategy in the treatment of T2DM patients.

## 2 Experimental design, materials and methods

### 2.1 Design, materials and methods

**Fig. 1:**
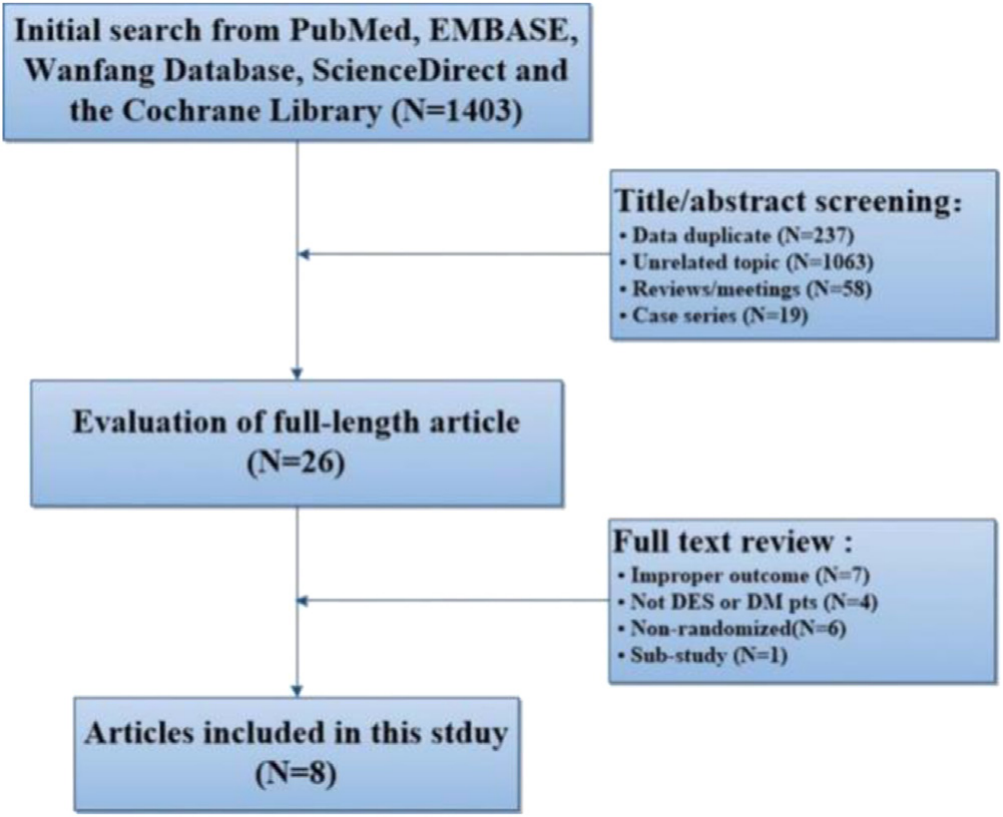
Flow diagram of the literature search process of this meta-analysis.

Relevant studies were identified from PubMed, Cochrane Library, Wanfang Database, Science Direct and Embase. The key words included cilostazol, stent, percutaneous coronary intervention and diabetes. A total of 1403 relevant publications were found in the initial internet retrieval. One of the articles [1] was excluded because it was a sub-study of another one [2] and was conducted by the same research group. Finally, eight randomized controlled trials (RCTs) [2], [3], [4], [5], [6], [7], [8], [9] met the inclusion criteria and were enrolled (Fig. 1). The dosage of cilostazol was 200 mg per day for 6 months. Data including the first author׳s surname, publication year, region, case number, gender, age, demographic data, target population, treatment protocol, follow-up period, efficacy outcomes and safety outcomes were extracted (Table 1).The primary efficacy outcome was MACEs which was defined as a composite of cardiac death, myocardial infarction (MI), stroke, target vessel revascularization (TVR), target lesion revascularization (TLR), or stent thrombosis.

**Table 1:** Main characteristics of all studies included in meta-analysis.

**Study**	**Country**	**Number Dual / Triple**	**Mean age (yrs) Dual / Triple**	**Male (%) Dual / Triple**	**Hypertension (%) Dual / Triple**	**Hypercholesterolemia (%) Dual / Triple**	**Current smoker (%) Dual / Triple**	**Multivessel disease (%) Dual / Triple**	**Follow-up (m) Dual / Triple**
Gao [9]	China	156/162	64.3/65.2	55.8/54.9	43.6/45.1	46.2/53.1	48.7/50.6	59.5/48.2	1
Lee [8]	Korea	84/92	62.1/60.9	71.5/70.0	64.7/58.4	45.0/42.4	30.1/30.4	37.3/34.8	12
Li 2010 [6]	China	30/30	NA	NA	NA	NA	NA	NA	9
Shen [7]	China	80/80	69.6/67.9	75/73.8	62.5/68.8	38.8/36.2	46.2/43.8	NA	12
Han [2]	China	122/141	59.6/59.5	77.0/71.6	72.1/70.9	55.7/53.9	43.4/44.7	72.1/80.1	12
Lee [5]	Korea	200/200	60.7/61.0	57.0/59.0	59.5/59.5	28.5/30.5	31.5/24.8	62.5/65.5	9
Lu [4]	China	79/78	61.0/61.0	NA	NA	NA	NA	NA	6
Lee [3]	Korea	81/85	61.2/60.9	63.6/64.8	55.2/54.8	28.4/30.0	37.2/37.6	59.6/66.8	9

NA: not available.

### 2.2 Meta-analysis

All statistical tests were performed with Review Manager 5.2 from the Cochrane Collaboration. Odds ratio (OR) with 95% confidence interval (CI) was used. The pooled OR was performed for dominant model. P value ≤0.10 was considered to be significant for statistical heterogeneity. Random-effect model was chosen in this study to reduce the potential bias. According to the funnel plot (Suppl. 1) and risk of bias graph (Suppl. 2), the reporting biases of this study was acceptable.

**Fig. 2:**
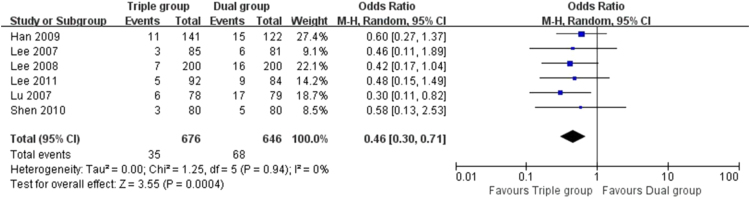
Forest plot of the risk of TVR. The risk of TVR in Triple group was lower than that of Dual group during a follow-up of 12 months after stents implantation.

**Fig. 3:**
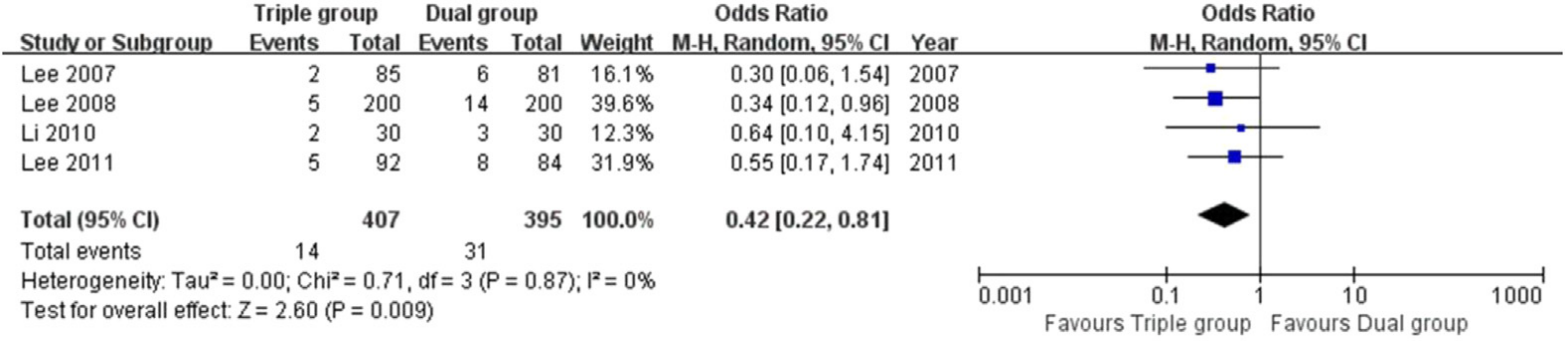
Forest plot of the risk of TLR. Compared to Dual group, the risk of TLR in Triple group was reduced significantly.

**Fig. 4:**
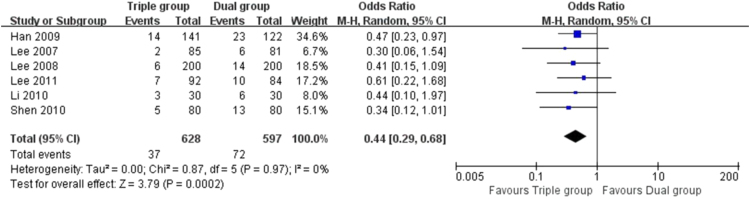
Forest plot of the risk of MACEs. Compared to Dual group, the risk of MACEs was reduced significantly in Triple group after stents implantation.

**Fig. 5:**
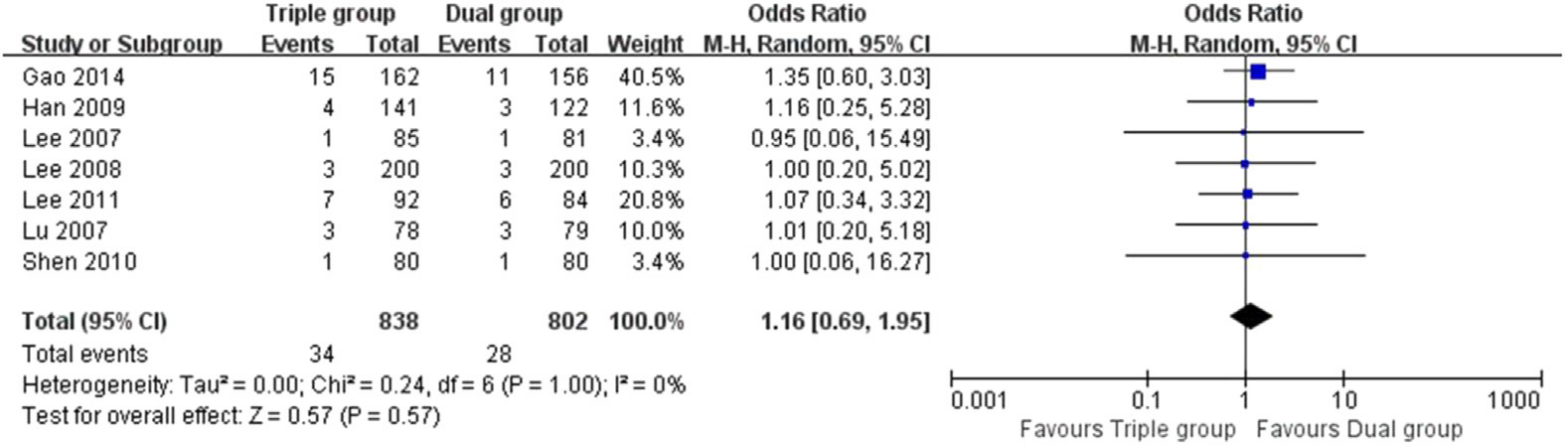
Forest plot of the risk of bleeding events. The risk of bleeding events were similar in Dual and Triple group during a follow-up of 12 months after stents implantation.

The data here showed that the risk of TVR (5.2% vs. 10.5%, OR 0.46 [0.30, 0.71], *P* = 0.0004, [Fig. 2]), TLR (3.4% vs. 7.8%, OR 0.42 [0.22, 0.81], *P* = 0.009, [Fig. 3]) and MACEs (5.9% vs. 12.1%, OR 0.44 [0.29, 0.68], *P* = 0.0002, [Fig. 4]) in Triple group were lower than that of Dual group. There was no significant difference in the comparison of stent thrombosis and bleeding events between the two groups (4.1% vs. 3.5%, OR 1.16 [0.69, 1.95], *P* = 0.57, [Fig. 5]).
