# Supplementary material for: An up-dated meta-analysis of major adverse cardiac events on triple versus dual antiplatelet therapy after percutaneous coronary intervention in patients with type 2 diabetes mellitus
Source: Data Brief. 2018 Jun 30;20:448–53. doi: 10.1016/j.dib.2018.06.091 (PMC6122306; doi:10.1016/j.dib.2018.06.091)
Supplement: Supplementary file 1 — Supplementary material [file mmc1.pdf]

ICMJE INTERNATIONAL COMMITTEE of MEDICAL JOURNAL EDITORS

# ICMJE Form for Disclosure of Potential Conflicts of Interest

## Section 1. Identifying Information

1. Given Name (First Name)
Lv

2. Surname (Last Name)
Zhan

3. Date
03-May-2018

4. Are you the corresponding author? ☑ Yes ☐ No

5. Manuscript Title
An up-dated meta-analysis of major adverse cardiac events on triple versus dual antiplatelet therapy after percutaneous coronary intervention in patients with type 2 diabetes mellitus

6. Manuscript Identifying Number (if you know it)
DIB-D-18-00305

## Section 2. The Work Under Consideration for Publication

Did you or your institution **at any time** receive payment or services from a third party (government, commercial, private foundation, etc.) for any aspect of the submitted work (including but not limited to grants, data monitoring board, study design, manuscript preparation, statistical analysis, etc.)?

Are there any relevant conflicts of interest? ☐ Yes ☑ No

## Section 5. Relationships not covered above

Are there other relationships or activities that readers could perceive to have influenced, or that give the appearance of potentially influencing, what you wrote in the submitted work?

☐ Yes, the following relationships/conditions/circumstances are present (explain below):

☑ No other relationships/conditions/circumstances that present a potential conflict of interest

At the time of manuscript acceptance, journals will ask authors to confirm and, if necessary, update their disclosure statements. On occasion, journals may ask authors to disclose further information about reported relationships.

## Section 6. Disclosure Statement

Based on the above disclosures, this form will automatically generate a disclosure statement, which will appear in the box below.

None.
