# Supplementary material for: An up-dated meta-analysis of major adverse cardiac events on triple versus dual antiplatelet therapy after percutaneous coronary intervention in patients with type 2 diabetes mellitus
Source: Data Brief. 2018 Jun 30;20:448–53. doi: 10.1016/j.dib.2018.06.091 (PMC6122306; doi:10.1016/j.dib.2018.06.091)
Supplement: Supplementary file 2 — Supplementary material [file mmc2.docx]

**An up-dated meta-analysis of major adverse cardiac events on triple versus dual antiplatelet therapy after percutaneous coronary intervention in patients with type 2 diabetes mellitus**

**Supplements**

**
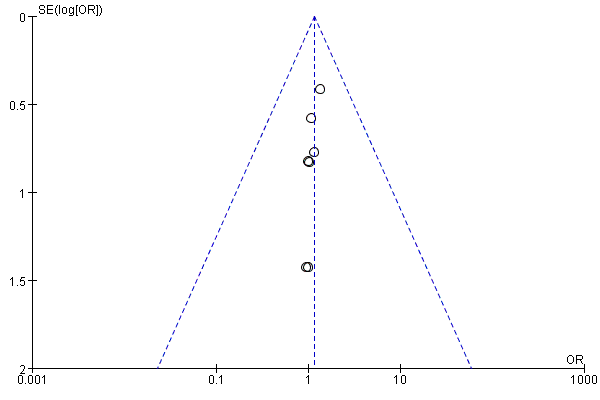
**

**Suppl. 1** The funnel plot of included studies


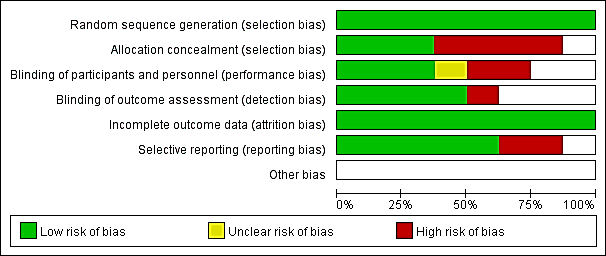


**Suppl. 2** The risk of bias graph of included studies
